# Applications of Deep Learning Algorithms to Ultrasound Imaging Analysis in Preclinical Studies on In Vivo Animals

**DOI:** 10.3390/life13081759

**Published:** 2023-08-16

**Authors:** Laura De Rosa, Serena L’Abbate, Claudia Kusmic, Francesco Faita

**Affiliations:** 1 Institute of Clinical Physiology, National Research Council (CNR), 56124 Pisa, Italy; laura.derosa@unitn.it (L.D.R.); francesco.faita@cnr.it (F.F.); 2 Department of Information Engineering and Computer Science, University of Trento, 38123 Trento, Italy; 3 Institute of Life Sciences, Scuola Superiore Sant’Anna, 56124 Pisa, Italy; serena.labbate@santannapisa.it

**Keywords:** review, preclinical model, in vivo animal model, deep learning, artificial intelligence, ultrasound imaging

## Abstract

Background and Aim: Ultrasound (US) imaging is increasingly preferred over other more invasive modalities in preclinical studies using animal models. However, this technique has some limitations, mainly related to operator dependence. To overcome some of the current drawbacks, sophisticated data processing models are proposed, in particular artificial intelligence models based on deep learning (DL) networks. This systematic review aims to overview the application of DL algorithms in assisting US analysis of images acquired in in vivo preclinical studies on animal models. Methods: A literature search was conducted using the Scopus and PubMed databases. Studies published from January 2012 to November 2022 that developed DL models on US images acquired in preclinical/animal experimental scenarios were eligible for inclusion. This review was conducted according to PRISMA guidelines. Results: Fifty-six studies were enrolled and classified into five groups based on the anatomical district in which the DL models were used. Sixteen studies focused on the cardiovascular system and fourteen on the abdominal organs. Five studies applied DL networks to images of the musculoskeletal system and eight investigations involved the brain. Thirteen papers, grouped under a miscellaneous category, proposed heterogeneous applications adopting DL systems. Our analysis also highlighted that murine models were the most common animals used in in vivo studies applying DL to US imaging. Conclusion: DL techniques show great potential in terms of US images acquired in preclinical studies using animal models. However, in this scenario, these techniques are still in their early stages, and there is room for improvement, such as sample sizes, data preprocessing, and model interpretability.

## 1. Introduction

Animal models are extensively used in biomedical research, with a broad spectrum of applications, ranging from basic science to the translation of methodological/technological enhancements for the clinical scenario. Indeed, the development of animal models of human disease has shown the potential in addressing questions about pathophysiological processes while maintaining the complexity of a whole organism. Further, translational models are currently used to achieve a more accurate classification and prediction and to evaluate novel diagnostic approaches and interventions. It is worth noting that the design of in vivo studies has been revolutionised by the advances of non-invasive imaging techniques and their application in preclinical models, offering the possibility to longitudinally monitor the same animal, with important implications in terms of data variability and the number of animals to study in experiments, as well as of enhanced information about disease progression and/or ageing.

The ever-increasing need for non-invasive techniques has assisted the rapid development of ultrasound (US) imaging, which is acquiring growing importance even in the preclinical field, both for its low cost and for its non-ionising nature compared to other modalities [1,2,3,4,5]. The US is now recognised as a valuable tool in the fields of oncology, cardiovascular medicine, and developmental biology. It has become the clinical standard for several procedures [6,7] and an important tool in the context of preclinical studies as well.

Since the early 2000s, manufacturers have provided US devices that apply to small whole animals to perform anatomical and functional imaging and the in vivo investigation of animal physiology and embryonic development. Since then, US applications in the preclinical setting have grown widely, with the development of scanners with enhanced temporal and spatial resolutions. To achieve adequate spatial resolution, small animals are imaged using ultra-high-frequency transducers, generally up to 20 MHz for rats, 40 MHz for mice, and more than 50 MHz for adult/embryo zebrafish and neonatal mice. Moreover, the improved temporal resolution of preclinical US systems led to the major challenge, the rapid heart movements observed in small animals (up to 600 beats per minute (bpm) in mice vs. 60–100 bpm in humans), being overcome [1].

US imaging has a variety of in vivo applications in animal models. One of the most common is echocardiography, for studying cardiac morphology and function in large-, medium-, and small-sized models of overt or subclinical cardiovascular disease [8,9,10]. Among the in vivo imaging modalities, functional US is widely applied to imaging brain function with very high spatial and temporal resolutions (in the order of microns and milliseconds, respectively). Such technological advancement led to the visualisation of even very small vessels, including the brain microvasculature [11].

Ultrasound localisation microscopy (ULM) allows for imaging microscopic vessels and measuring blood flow in the brain with very high spatial resolution and depth of penetration. It works by using microscopic bubbles circulating in the bloodstream as a contrast agent to measure the reflection of high-frequency acoustic waves passing through the body during US imaging acquisition [12,13,14,15].

In addition, photoacoustic (PA) imaging is emerging as a modality to study blood vessels in preclinical studies (as well as in the medical area) [16,17]. PA is based on imaging through the acoustic detectors of signals emitted by tissue components in response to optical excitation, and it provided high-level results in several brain studies [18,19,20,21].

In addition to the reconstructed final images (acquired in the B-mode/M-mode/A-mode/colour Doppler modalities), some advanced US machines also provide access to raw radio frequency (RF) signals. RF signals carry valuable information about acoustic wave propagation and its tissue interaction, thus providing the data to characterise the tissues and organs under study [22]. Elastography is an example of a US-based application that uses RF raw data and performs the analysis of tissue deformation following the application of stresses (i.e., manual, natural, and acoustical) to obtain the measures of mechanical parameters [23].

Still, despite the potential of US imaging, open challenges remain for its application in healthcare, including low sensitivity and specificity and operator dependence. In recent decades, to overcome these limitations, artificial intelligence (AI) has become increasingly widespread in the field of US image processing.

In this scenario, the available literature on the use of AI in US imaging highlights the current application of different techniques, from machine learning (ML) to deep learning (DL), demonstrating a significant advance in US imaging for acquiring and processing data. DL, nowadays, represents state-of-the-art ML methods in a variety of application areas; worth noting, it has emerged as a powerful tool in medical imaging. DL is a type of representation learning approach that uses complex multi-layer neural network architecture to automatically learn data representations by transforming the input information into multiple levels of abstraction using non-linear modules [24]. DL methods have remarkable performance compared to conventional ML due to the very high amount of data in DL model training.

DL techniques are developing very fast and for many tasks, from segmentation and/or classification tasks to the detection of specific patterns in images or feature extraction, to solving more acquisition-related problems, such as improving image quality, deleting electromagnetic noises due to the machines and performing real-time US beamforming.

Convolutional neural networks (CNNs) have assumed great importance among the several DL-based architectures proposed over the years. A CNN is a neural network specialised for working with images as input information. The most famous CNNs are LeNet, AlexNet, ResNet, GoogleNet, MobileNet, VGG, and U-Net [25]. Different from CNNs, to propose systems able to work with time series data or data that involve sequences, recurrent neural networks (RNNs) have also been developed [26].

Over the last decade, the introduction of DL methods in US imaging continues to elicit considerable interest in a variety of research areas. Here, we provide a comprehensive overview of the employment of DL techniques in US analysis in in vivo animal models as a useful experimental context to discuss the challenges and opportunities of their application in healthcare. In the next section, we provide a general description of the methods and output of the selection process. In Section 3, we analysed recent evidence about the role of DL techniques in US imaging by discussing their applications according to the targeted organs, including both major (cardiovascular, abdominal, musculoskeletal, brain) and minor (tumour vasculature, lymph nodes, embryos) anatomical districts. A conclusive section is then provided to discuss the frontiers and challenges of DL application to preclinical US in the healthcare field.

## 2. Materials and Methods

In this section, we described the search strategy adopted and explained in detail all the inclusion/exclusion criteria that led to the collection of the final papers object of the review.

### 2.1. Data Sources and Searches

We ran a literature search to identify all the relevant articles on the use of DL techniques applied to US imaging in preclinical in vivo models. We systematically searched PubMed/Medline and Scopus databases in the decade from January 2012 to November 2022. A systematic review was performed according to the Preferred Reporting for Systematic Reviews and Meta-Analysis (PRISMA) guidelines [27]. We performed advanced research by concatenating terms with Boolean operators. In particular, the search strategy included a combination of the following terms: (“fish” OR “sheep” OR “non-human primate” OR “porcine” OR “swine” OR “rodents” OR “veterinary” OR “rat” OR “pig” OR “animal” OR “mice” OR “preclinical” OR “dog” OR “mouse” OR “rabbit”) AND (“deep learning” OR “deep-learning” OR “neural network” OR “neural networks” OR “CNN” OR “convolutional neural network” OR “UNet” OR “U-Net” OR “artificial intelligence”) AND (“ultrasound” OR “echography” OR “sonography”). No date or language filters were employed in the initial search. The literature search was performed and verified by two authors (L.D.R., S.L.A.).

### 2.2. Eligibility Criteria

The inclusion criteria were:

- Studies on preclinical/animal models with in vivo US acquisitions and developed or tested DL-based algorithms on US images or features extracted from the images;
- No restriction on the animal species used;
- No restriction on the DL architecture adopted in the studies and/or on their tasks;
- Studies using in vivo preclinical US images only for testing DL model performance.

The exclusion criteria were:

- Studies performing US acquisitions on phantoms/ex vivo models/humans only;
- Studies proposing AI-based methods but not properly deep architectures;
- Publications not in the English language;
- Non-peer-reviewed original articles or conference proceedings.

Furthermore, the following publication types were excluded: reviews, conference abstracts, conference reviews, short communications and book chapters.

### 2.3. Data Extraction and Analysis

Two investigators (L.D.R. and S.L.A.) screened the articles separately. Disagreement between reviewers was resolved by consensus via discussion and checked by a third reviewer (F.F.). Reasons for the exclusion of some studies are better detailed in the Results section. Publications by the same research group or by different groups using the same dataset or DL models were included in the analysis. After selecting the articles, we collected the following features: first author’s surname and year of publication, animal model used, anatomical district under study, the aim of the study, task of the proposed DL network, DL architecture used, and main results obtained.

## 3. Results

### 3.1. Search Results

**Figure 1:**
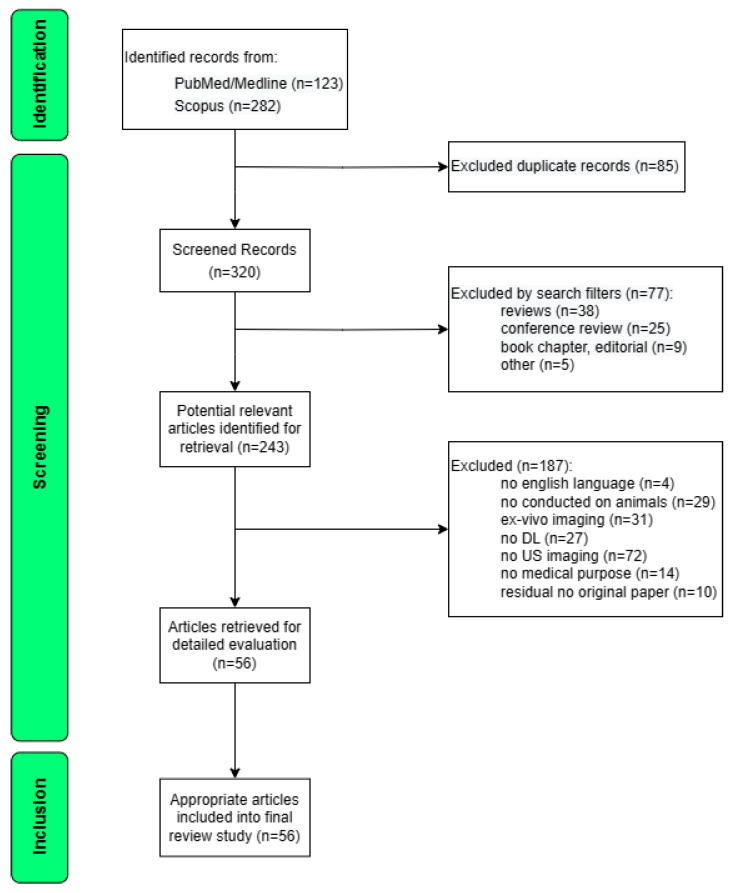
Flow diagram of systematic identification, screening, and inclusion of articles on the use of DL techniques applied to US imaging in preclinical in vivo models. DL: Deep learning; US: Ultrasound.

The literature search revealed 405 publications as the total number of papers output from both databases used in the study; after excluding 85 duplicates, 320 records were screened. Then, by filtering out the 77 papers that included conference reviews, reviews, book chapters and short communications, 243 papers were selected. Following the review of the title and abstract and, upon necessity, the full text further 187 records were rejected. After the reviewing process, a total of 36 original papers [28,29,30,31,32,33,34,35,36,37,38,39,40,41,42,43,44,45,46,47,48,49,50,51,52,53,54,55,56,57,58,59,60,61,62,63] and 20 conference proceedings [64,65,66,67,68,69,70,71,72,73,74,75,76,77,78,79,80,81,82,83] met the inclusion criteria. Figure 1 illustrates the flow chart of identification screening and selection processes.

Table 1 and Table 2 show the main characteristics of the included original papers and conference proceedings, respectively.

**Figure 2:**
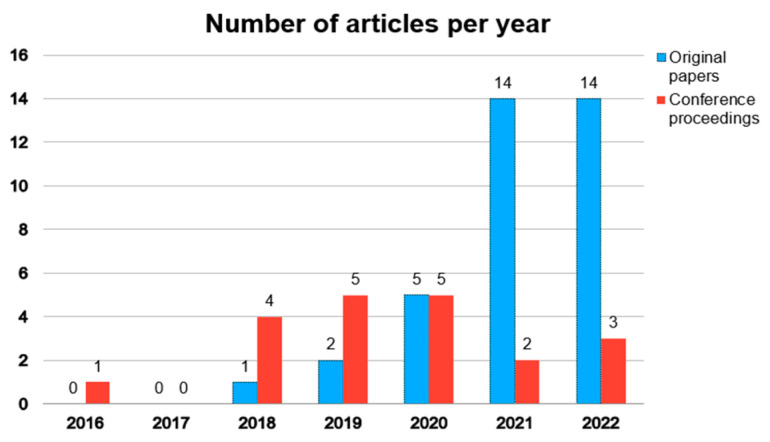
Temporal distribution of the 36 original articles and the 20 conference proceedings based on the year of publication.

Although the search strategy included articles published within the decade 2012–2022, almost all selected papers were published in the last five years (Figure 2).

**Figure 3:**
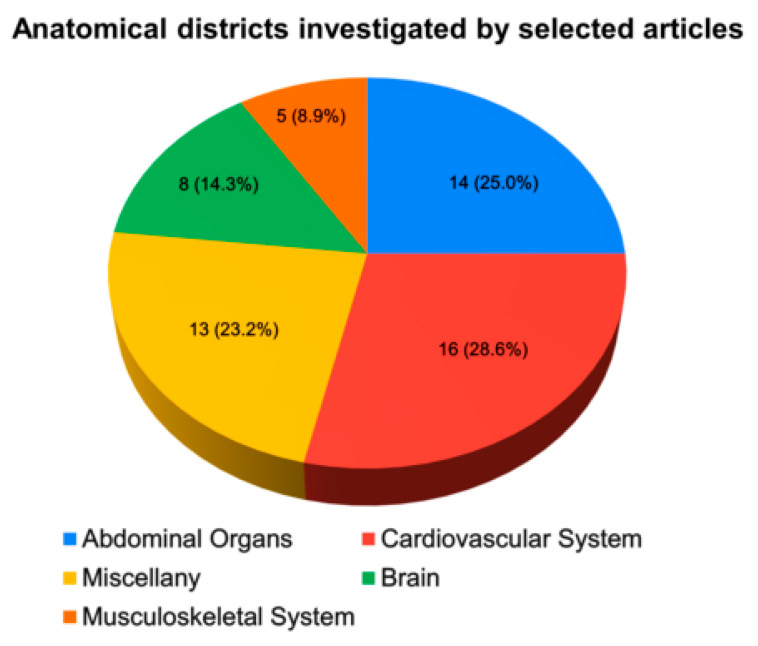
Pie chart representing the partition of publications according to the number of publications, and their relative percentage in parenthesis, based on the anatomical district in which DL models were used on US imaging analysis.

In the upcoming subsections, we will propose an analysis of the papers by categorising them based on the anatomical region in which DL models were applied to US images (Figure 3).

### 3.2. Cardiovascular System

US imaging techniques are widely used to investigate the cardiovascular system, e.g., to study heart function/morphology, to track blood flow and for early identification of any functional impairment. Among the 56 selected papers, 16 (28.6%) proposed the application of DL to US images in cardiovascular studies. In particular, these papers focused on the heart [43,45,48,54,64,73,83], atherosclerotic plaque [34], carotid [56]/abdominal [40]/femoral [31,52,75] arteries, femoral veins [65,66] and inferior vena cava [77].

Two papers [34,40] used rabbits in their experiments. Cao et al. [34] proposed a network for studying the severity of atherosclerotic lesions aiming at classifying the vulnerability index assessed by the images of the plaque. On the other hand, in reference [40], the DL network was validated using images from both phantoms and in vivo animals. The purpose was to localise the microbubbles injected into the abdominal artery and distinguish between the signal emitted by the microbubbles and the signal generated by the tissue.

Nine papers [31,48,52,54,65,66,73,75,77] dealt with porcine models. In all these studies, the number of enrolled animals was very low (1, 10, 11, 7, 1, 1, 1, 5 and 2 pigs in [31,48,52,54,65,66,73,75,77], respectively). The small sample size of studies on medium-sized animals could be partly due to the challenges involved in managing experiments, such as costs and housing spaces, equipment, etc., despite the cardiovascular anatomy and physiology of pigs being closer to that of humans [84]. In [52], a deep convolutional generative adversarial network (GAN) was designed to explore blood flow anomalies associated with haemorrhage on US colour Doppler images acquired from femoral arteries. Haemorrhages were detected with an area under the receiver operator curve (AUC) of 0.90, 0.87, and 0.62 (immediately after, 10 and 30 min post-injury, respectively). Studying the femoral vasculature, Brattain et al. [31] proposed a DL network for detecting needles during femoral vascular access with a precision of 0.97 and 0.94, and a recall of 0.96 and 0.89 for artery and vein, respectively.

Murine models have been adopted in four papers [43,45,56,83]. Park et al. [56] proposed a U-Net modified network to study flow–vessel dynamics in the carotid artery; they also demonstrated that the DL model performed better than the conventional US-based flow and strain measurement techniques in assessing vascular stiffness.

In [45], a classification network for differentiating between normally perfused and infarcted myocardial regions was proposed. The system achieved high classification precisions of 99.6% and 98.7% and an AUC of 0.999 and 0.996 on two different test sets, respectively.

Seven papers [43,48,54,64,73,77,83] proposed DL models designed for segmentation tasks. Three of them focused on the left ventricle [43,48,64], one at segmenting the whole heart during cardiac arrest [54] and another on segmenting both the epicardium and endocardium [83]. In [73], the aim was to segment a guidewire in real-time during a cardiac intervention, and in [77], the authors proposed a system to identify vessel lumen. In particular, Duan et al. [43] proposed a fully automated tool named mouse-echo neural net, in which a deep CNN (U-Net-based architecture) was implemented to perform semantic segmentation on both B-mode (to locate left ventricle borders) and M-Mode (to identify anterior/posterior walls, LV and background) images. The automatic segmentation achieved a very high dice similarity coefficient (DSC) of 92.45% and 95.63% compared to the manual segmentation of B-Mode and M-Mode images, respectively. These results were consistent with those obtained via manual analysis. In [48], the authors applied U-Net and a GAN model for LV cavity segmentation, achieving very similar results, with a DSC of 0.90 and 0.91 for U-Net [25] and GAN model [85], respectively. Interestingly, in [54], the authors fed an AI-based bladder scanner designed to segment the bladder with heart images to evaluate its performance in the segmentation of the left ventricle. They achieved very promising results, finding that this device was able to identify cardiac arrest with high reliability by tracing the borders of the heart in a pig model.

### 3.3. Abdominal Organs

The US is widely adopted for investigating organs and soft tissues in the abdominal cavity. Indeed, 14 (25%) of the reviewed papers [29,37,44,47,49,50,53,60,62,67,69,72,76,81] have been focused on abdominal US imaging. In particular, studies focused on liver, kidney, spleen and bowel examinations.

Eleven papers focused on liver analysis [29,37,49,50,53,60,67,69,72,76,81]. In [49] and [72], the same DL network was used to segment the liver, brain and kidney. The authors proposed a U-Net-modified network able to work with hybrid optoacoustic (OA) and US images (dual-modality) acquired in mouse models. A preliminary study has been conducted in [72], a conference proceedings publication in which the authors trained a CNN-based network only on OA images and then tested it on both OA and US images. Subsequently, in [49], the authors investigated more in-depth their proposed system. Interestingly, they trained their model by mixing images acquired from the liver, brain, and kidney to demonstrate the robustness of the network in segmenting different organs with different outlines and contrast. They achieved good results in terms of the DSC index (0.76), outperforming a traditional segmentation technique. This finding shows the translational ability of DL models from one organ to another. U-Net trained on two techniques (OA + US) showed lower performance compared to the model trained with OA images alone. The authors justified the lower performance achieved by the dual modality with the lower availability of US data relating to OA data.

In [50], the authors proposed a deep CNN with multi-feature extraction applied to B-mode rat liver images. By integrating some information extracted from the parametric maps provided by the US device, they obtained a sensitivity of 0.82, specificity of 0.84, accuracy of 0.83 and AUC of 0.87 in the recognition of significant liver fibrosis on the test set data. These results were comparable with those obtained with the validation set data.

Eight papers [29,37,47,53,60,67,76,81] proposed DL models for classification tasks in organ disease. In particular, Banzato et al. [29] developed a deep neural network using transfer learning (AlexNet retrained and fine-tuned) for the diagnosis of degenerative liver disease in dogs. In [81], the authors proposed an RNN (LSTM-based network) to classify liver fibrosis stages (S0-S4) using 96 RF signals acquired (80/16 in training/validation, respectively). Later, the same group proposed a bidirectional long short-term memory to classify the severity of rat hepatic fibrosis according to five score classes [37] by increasing the number of data (160 RF signals acquired from 33 rats) and implementing the network architecture regarding [81]. This is worth noting that the models showed a better performance in [37] concerning [81] in the validation sets for classifying <S0/<S1/<S2/<S3. The former study achieved AUCs of 0.93/0.95/0.98/0.99 compared to values of 0.90/0.94/0.92/0.93 in the latter investigation. This slight improvement in the performance may be ascribed to the increased number of data used in reference [37] for training the networks. Moreover, in four papers, the authors proposed CNN models to classify in the rabbit the severity of liver fat content [53,67,76] and fibrosis [60]. Finally, Jiang et al. [47] applied MobileNetV2 for 2D feature extraction, followed by ResNet models for the classification of splenic trauma in pigs. In the papers [37,50,60], all the authors proposed DL systems to classify the stages of liver fibrosis. It is noteworthy that the results achieved were very similar despite the use of different architectures and strategies. Accuracies greater than 0.8 and AUCs between 0.82 and 0.95 were reached in all cases, particularly in the detection of significant stages of liver fibrosis.

In the conference proceedings [69], the authors studied the perfusion of hepatocellular carcinoma through microbubble detection in a mixed dataset composed of US molecular images acquired in a mouse model and phantoms. They achieved a significant improvement in image reconstruction concerning conventional beamforming methods with an AUC of 0.90 in microbubble detection into the mouse tumour.

In addition, two papers [44,62] described CNN architectures aimed at improving the quality of US images by removing electrical noise. Both papers tested the proposed DL models on endoscopic PA/US images of the bowel, namely the rat colon–rectum and the rabbit urinary tract [44] and the rabbit rectum [62].

### 3.4. Musculoskeletal System

Despite US imaging being widely used to study the musculoskeletal system in clinical studies, there is still only a partial exploration of this imaging modality in preclinical investigations. Indeed, only five [35,38,42,55,61] papers (8.9%) proposed deep learning architectures to work with US images of bones [38,61], muscles [35,55] and teeth [38,55].

Pig is the preclinical model chosen in the majority of the papers in this section [35,38,55], and only two papers proposed studies on rats [42] and rabbits [61].

All these papers [35,38,42,55,61] trained DL systems for segmentation tasks and all of them proposed U-Net-based models properly adapted for the specific training tasks. In [35], Carson et al. proposed a system able to detect, segment, classify, and display neural structures during trans-psoas spine surgery by processing B-mode images. The authors integrated a U-Net in their AI system to classify bone and muscle regions in B-mode images surrounding the muscle psoas. A U-Net modified (called ResTU-net) has been proposed in [42] for the segmentation of muscles (gastrocnemius and soleus) in the rat hindlimb. The performances DSC of 94.82% and 90.72% achieved for gastrocnemius and soleus, respectively, outperformed the state-of-the-art methods. In [55], a multi-class deep learning segmentation system based on a U-Net was designed and trained on 274 premolar sonograms (including augmented data) acquired from five pigs. This network was able to automatically identify several of the dental and periodontal structures (e.g., alveolar bone, gingiva and oral mucosa, and crown) in each image. Concerning tooth, gingiva and mouth bones, in [38], the authors developed a model for the 3D reconstruction of those structures using high-frequency US (HFUS) images acquired with a free-hand 2D system equipped with a spatial positioning reading sensor. For this purpose, a network previously proposed (Mask R-CNN [86,87]) was retrained to automatically segment tooth, bone and gingiva.

Finally, Tang et al. [61] proposed a DL segmentation network (based on U-Net) for segmenting the spine surface using a fusion of US and computed tomography (CT) images. The proposed 3D reconstruction method would allow its use during spinal intraoperative sessions without the need for an external tracking system.

### 3.5. Brain

Eight papers [30,39,41,51,59,63,68,70] proposed DL models applied to US images of the brain. Seven of them focused on the study and visualisation of brain microvasculature [30,41,51,59,63,68,70] and one [39] on the improvement of image quality using technologies of image fusion (PA and CT). These papers applied the DL approach to mice [30,63,70] and rats [39,41,51,59,68].

Milecki et al. [51] validated their DL system on images acquired from one rat only. They trained a 3D-CNN based on a V-net architecture (named Deep-stULM) in brain perfusion analysis through microbubble localisation by using a dataset composed of in silico simulated mouse brain microvascular networks. The authors commented on how their network performed and generalised well over an in vivo dataset without providing any statistical demonstrations.

Six [30,39,59,63,68,70] papers aimed at using DL techniques to improve the quality of the US microvascular images. Blons et al. [30] proposed a DL model called PerfectFlow (U-Net modified + VGG16-derived) that used a perceptual loss function to enhance the visualisation of brain microvessels in transcranial Doppler images of mouse brains. Alike, in [39], the authors proposed a DL architecture, called a 3D progressive U-shaped enhancement network, trained on fused photoacoustic computed tomography (PACT) and US images. The network’s task was to improve the quality of PACT images, a practice that is now widely used in preclinical settings. In [59], the authors compared the performances of a convolutional robust principal component analysis network, which is a fixed-length deep network, with a conventional ResNet in the representation of the vasculature. In [63], Zhu et al. developed a DL method (termed fully dense U-net) to dampen the discontinuity/low resolution associated with the subsampling of cerebral microvasculature images acquired with an ultrafast functional PA microscopy (UFF-PAM) system. Their model was trained on fully sampled images to recognise and reconstruct microvessels via oversampling. Once trained, a fully dense U-net was applied to their sub-sampled images acquired with UFF-PAM. The end outcome was an improvement in spatial resolution and a clearer visualisation of the cerebral microvascular network of the mouse. In [70], the authors proposed an FCN-based beamforming technique on 3D ULM images, obtaining a significant improvement in image reconstruction concerning conventional beamforming methods. Even Cohen et al. [68] reported an improvement in vasculature visualisation of the rat brain by using DL systems compared to common methods. Specifically, they trained an unfolded network solely on simulated data and tested the performance on in vivo images.

Instead, Di Ianni et al. [41] compared the performances of their network (Deep-fUS) based on a 3D-Res-U-Net with those of a simple U-Net on images of the brain microvasculature of rats. Deep-fUS achieved better performance in the reconstruction of power Doppler images, with values of the peak signal-to-noise ratio of 28.8, normalised mean square error of 0.05 and a mean absolute error for the activation map of 0.1193 lower than that obtained by simple U-Net and with a compression factor of 85%.

Interestingly, all papers presented in this section proposed DL networks to improve brain microvessel detection compared to traditional approaches, albeit using different DL architectures and US technologies. In all these articles, the application of the DL methods provided a significant improvement in the image quality of the visualised cerebral vessels, both in terms of better spatial resolution and reduction in artefacts and errors.

### 3.6. Miscellany

This last paragraph collects 13 papers [28,32,33,36,46,57,58,71,74,78,79,80,82] that proposed heterogeneous US-based applications adopting DL systems. In particular, papers focused on embryo segmentation [28,57,79,80,82] and embryo reconstruction [78] or on the vascularisation of breast cancer tissue [32,46], lymph node [58], hind limb [33], chorioallantoic membrane [36] and lung [71,74].

Nine papers [28,32,33,46,57,58,79,80,82] adopted murine models, two [36,78] were working with chicken US images and two [71,74] with pig models.

Five of the selected papers [28,57,79,80,82] have been proposed by the same research group that provided DL-based systems for 3D body and brain ventricle segmentation in mouse embryos. First, in a study published in [80], the authors developed a framework based on 3D-CNN for segmenting brain ventricles. They achieved a DSC of 0.896 by testing the model on 111 HFUS images. In a second study [79], Qiu et al. incorporated their framework with an embryo body segmentation tool which resulted in a higher DSC of 0.925. In addition, they developed a classification model for distinguishing between normal and mutant embryos. An additional optimisation of their framework was presented in [82], in which the authors reached a very similar level of network accuracy but with a significant decrease in inference time (about 1000×). All these findings were assembled in two original articles [28,57]. Indeed, in [57], authors comprehensively described the previously proposed framework, and in [28], they tested it again on an external dataset that included images acquired in nine pregnant mice/101 embryos.

In [32], the authors proposed a DL-based spatiotemporal filter for microbubbles images formation and segmentation of in vivo super-resolution US images in a murine model of breast cancer. Their 3D-CNN achieved 84.3% accuracy, 84.7% sensitivity and 83.8% specificity in the in vivo training of the network. Additionally, an optimised version of the previously mentioned DL model was applied in [33] to perform contrast agent detection and localisation in studying the rat hind limb vasculature images. Their network was created from blocks of the MobileNetV3 architecture customised for 3D data. In a similar task, Hyun et al. [46] proposed a fully convolutional neural network to study breast tumour vascularisation in a mouse model by US microscopy image processing, thus obtaining a DSC of 0.45 and AUC of 0.91.

Sharma et al. [58] developed a network to improve the quality of PA microscopy images of the vasculature around the sentinel lymph node in a murine model. They designed a fully dense U-Net that improves resolution and signal strength while reducing background signal.

Two papers [71,74] focused on lung US examination in pigs. In both articles, a CNN-based network was used to analyse swine US videos. In [71], the authors proposed an Inception V3-based CNN for detecting and classifying five lung abnormalities. Then, Mehanian et al. [74] applied the previously proposed method in pneumothorax detection. They also proposed an RNN (based on the LSTM network) to perform temporal analysis and achieve better performance in the automatic detection of the absence of lung sliding.

Finally, Chen et al. [36] proposed a neural network for microbubbles localisation microscopy aimed at the real-time visualisation of the high-resolution microvasculature of the chicken embryo chorioallantoic membrane. The network has been previously trained on simulated data, and the images acquired in vivo were used only during the testing phase.

## 4. Discussion

DL has recently emerged as an alternative approach to dealing with the limitations of US image analysis (such as operator dependence), and its application to clinical in vivo US is gaining popularity in various research fields. The integration of DL architectures into the preclinical US might represent a valuable tool in experimental studies and a step towards their adherence to 3R strategies. However, this approach is still evolving and relatively new in in vivo animal models. In this review, we offered an overview of the applications of DL techniques in preclinical US imaging on in vivo models. We searched for articles published from 2012 onwards; our selection criteria only allowed for the collection of publications starting from 2018 (except for one conference paper published in 2016), with a significant increase in the number of articles in the last 2 years. Evidence indicates that the use of DL systems in preclinical US imaging has been recently introduced and that the application of this approach is rapidly growing in a variety of in vivo models.

Our analysis revealed that murine models (i.e., mice and rats) are used in the majority of in vivo studies applying DL to US imaging (52% of the selected articles). This reflects the prevalent use of rodents in in vivo studies, and it is likely attributable to their low costs, ease of handling, and, notably, their relatively short lifespan and reproductive cycle. These characteristics make them a valuable model for a plethora of research applications. Our analysis has identified a considerable variety of US-based DL applications with different tasks, such as the segmentation of organs, disease severity classification, image quality improvement, and contrast/microbubbles localisation for studying blood flow and microcirculation. A lower number of studies used porcine models (27% of the reviewed papers) that are particularly suited for applications requiring a close similarity to human anatomy, despite their costs in terms of purchase and maintenance. Among the reviewed studies in porcine models, DL has been prevalently applied for muscle/bone structure segmentation or for testing US-guided interventions. Rabbits were used in 9 of 56 articles as an intermediate model between small and medium-sized animals. In these studies, DL was applied on US images either for cardiovascular applications, including the classification of plaque vulnerability and vascular localisation of microbubbles and for the classification of liver steatosis and fibrosis.

It should be underlined that, despite the recent growth of DL applications in preclinical imaging, there is still a substantial difference between the clinical and preclinical application fields concerning the smaller number of image samples in the latter. Indeed, many in vivo animal model studies use a low number of data in training the networks, often without rigorous validation, thus limiting the ability to generalise to newly acquired data never seen by the network [88].

The studies analysed showed that the networks were trained with a limited number of images, and the animals from which the images were obtained were also very heterogeneous and variable in number. Only seven articles used more than (or equal to) fifty animals (52 dogs, 50 pigs, 653 mice, 84 and 96 rats, 57, and 80 rabbits in [29,35,43,50,53,60,81], respectively). Moreover, nine papers [36,57,63,67,69,79,80,82,83] did not report the number of animals enrolled. In the study by Duan et al. [43], which included more than 600 mice, the authors propose an automatic tool for the rapid analysis of B-mode and M-mode images, within which the first step of segmentation was managed by a U-Net network. This approach resulted in a significant reduction of over 92% in the time taken for image analysis. In addition, there were excellent correlation coefficients (ranging from 0.93 to 0.98), and automated and manual segmentation showed good agreement. Furthermore, greater accuracy of the analysis was found due to the reduction in operator-dependent variability.

The availability of large and shared datasets is one of the major challenges for the widespread use of DL systems in preclinical ultrasound imaging. A large training sample with an accurately verified reference standard is mandatory for developing a well-performing DL model. However, due to the current limited availability of large datasets, the transfer learning (TL) technique is commonly used to overcome these problems. It consists of the use of pre-trained networks on large datasets (e.g., ImageNet [89,90]) and then fine-tuning them on a small number of new input data.

Another commonly used strategy to increase the number of data and minimise overfitting is the data augmentation approach. A series of basic transformations (e.g., rotations, zooming/scaling, *x*- and *y*-axis movement) are applied to the data to generate modified copies of it to be used in the model training. However, it should be noted that performing data augmentation is not properly equivalent to an increase in new and independent data, and often neural networks do not benefit from the excessive addition of augmented data [88]. Alternatively, more complicated techniques are also developing, such as Generative Adversarial Networks (GANs), which generate plausible new data compared to the available ones [88]. The majority of the selected papers [28,29,30,31,32,33,34,36,37,38,41,42,45,47,48,49,50,57,58,61,71,72,73,74,77,78,79,80,82] used TL techniques to overcome data limitations rather than developing models from scratch. Data augmentation was performed in twenty-eight of the analysed papers, dealing with the cardiovascular system [31,34,48,56,73,75,77], abdominal organs [29,44,49,50,60,72,81], musculoskeletal system [55,61], embryo [28,57,78,79,80,82], breast tumour vascularisation [32,46], brain [59,63] and lung [71,74] US images. In addition, GAN models have been applied in three papers [48,52,75].

The studies reported in [33,51] utilized a different approach. Brown et al. [33] performed testing of a CNN using images of the rat hind limb acquired in vivo. This CNN, however, was trained on in silico data. Milecki et al. [51] proposed a 3D-CNN model trained on in silico ULM simulated data and validated on rat brain images acquired in vivo. Both the studies achieved promising results and demonstrated the generalisation capability of their models, as well as the translatability of DL applications trained on simulated data in reproducing comparable results on real images.

The high computational power required is another important aspect in the development of DL systems due to the large number of data involved in the process of training DL models. Therefore, high-performance graphics processing units (GPUs) with plenty of memory available are required to handle large volumes of calculations/operations.

It is worth mentioning that the standardisation of quantitative indicators and benchmarking techniques is a critical aspect when evaluating the effectiveness of proposed methods. Indeed, the selected studies had very heterogeneous objectives (Table 1 and Table 2, Main Results column), ranging from the activity of classification to segmentation, extraction of image characteristics or improvement of the quality of the image. Consequently, the criteria used to evaluate performance were also quite heterogeneous and varied: e.g., the DSC for the evaluation of DL-model segmentation performance compared to the manual segmentation or the accuracy/specificity/sensitivity for assessing the performance for classification tasks, the quantitative signal-to-noise ratio to assess the image quality or more specific indexes of performance for specific tasks. This variety made it challenging to compare and comment on the numerical results presented by the authors.

Despite the current circumstances, it is highly recommended to enhance the development and implementation of DL algorithms in preclinical US data analysis. The results obtained so far seem encouraging. Preclinical US imaging, with its different modalities and applications, can serve as a great platform for developing and testing DL systems on translational model images of human diseases. These models range from small animals to those more similar in size to humans. Therefore, the implementation of high-performing DL models, along with their validation in preclinical studies, can represent an added value when successfully imported into medical imaging.

## 5. Conclusions

The use of DL methods with US imaging in medical imaging has recently gained attention, but its application in preclinical in vivo studies is still in its early stages. This paper aimed to systematically review the literature to determine the potential validity of DL-based systems for US preclinical data analysis. In preclinical studies, there is a high priority for the role of DL to automate complex tasks (e.g., quantification, segmentation, reconstruction) or improve image quality (e.g., dose/noise reduction). There are still some weaknesses that prevent the widespread use of DL models, such as the need to collect large numbers of samples and the requirement for more rigorous and standardised approaches to compare the models used in different studies. The implementation of these technologies in preclinical biomedical science is highly advisable, as they can provide a vast amount of information through animal models that mimic human pathophysiology or clinical scenarios. In perspective, well-trained and tested DL algorithms developed on preclinical US imaging can potentially be imported as new diagnostic/prognostic tools in the medical field.

## Figures and Tables

**Table 1:** The main characteristics of the original articles included in the analysis.

Ref	Animal Model	Anatomical District	Aim of Study ^1^	DL Network Task ^1^	DL Architecture ^1^	Main Result ^1^
[28]	mouse	Embryo	Segmentation of Embryo body	Segmentation	FCN	no significant changes between control and mutant mice embryos
[29]	dog	Liver	Binary classification of degenerative hepatic disease	Classification	DNN	AUC = 0.91; Se = 100%; Sp = 82.8%; PLR = 5.25; NLR = 0.0
[30]	mouse	Brain Vasculature	Vessel visualisation improvement	Image Quality Improvement	CNNs	CNR ↑ 56%; spatial resolution ≃ 100µm
[31]	pig	Femoral Artery	Needle detection to create femoral vascular access	Needle Detection	CNN	Precision = 0.97–0.94; Recall = 0.96–0.89 in artery and vein detection, respectively
[32]	rat	Breast Tumour Vasculature	MB segmentation and localisation through a spatiotemporal filter	MB Localisation	3D-CNN	Acc = 88.0% Se = 82.9% Sp = 93.0%
[33]	rat	Hind Limb Vasculature	Tissue decluttering and contrast agent localisation	Contrast Agent Localisation	3D-CNN	Qualitative results
[34]	rabbit	Plaque	Classification vulnerability of atherosclerosis plaques	Classification	CNN	AUC = 0.714; Acc = 73.5%; Se = 76.92% and Sp = 71.42%
[35]	pig	Psoas Muscle	Classification of bone and muscle regions	Segmentation + Classification	CNNs	DSC = 92%; Acc > 95% for nerve detection; DSC > 95% for bone and muscle
[36]	chicken	Embryo Chorioallantoic Membrane	MB localisation for real-time visualisation of the high-resolution microvasculature	MB Localisation	CNN	faster localisation than the conventional method to reach 90% vessel saturation; >20% faster than MB separation
[37]	rat	Liver	Classification of liver fibrosis severity (F0-F4)	Classification	RNN	Acc = 0.83–0.80; AUC = 0.95–0.93 in train and validation tests, respectively
[38]	pig	Tooth, Bone and Gingiva	Segmentation and 3D reconstruction	Segmentation	CNN	mean accuracy precision (mAP) > 90%
[39]	rat	Brain and Whole Body	Improvement of image quality using image fusion (PA + CT)	Image Quality Improvement	3D-CNN	↑ static structural quality/dynamic contrast-enhanced whole-body/dynamic functional brain acquisitions
[40]	rabbit	Abdominal Artery	Differentiation of MB from tissue on RF signals	MB Localisation	CNN/RNN	↑CTR and CNR by 22.3 dB and 42.8 dB, respectively
[41]	rat	Brain Vasculature	Brain vasculature reconstruction	PD Reconstruction	3D-CNN	PSNR = 28.8; NMSE = 0.05 and MAE = 0.1193, with an 85% compression factor
[42]	rat	Shank Muscle	Segmentation of the shank muscle	Segmentation	CNN	DSC = 94.82% and 90.72% for Gas and Sol muscles, respectively
[43]	mouse	Heart Left Ventricle	Segmentation of left ventricle	Segmentation	Deep CNN	time analysis reduction > 92%; Pearson’s r = 0.85–0.99
[44]	rat and rabbit	Colorectum and Urethra	Removing EMI Noise	Image Quality Improvement	CNNs	U-Net modified outperforming in EMI noise removal vs. others
[45]	mouse	Heart	Identification and classification of myocardial regions (health/infarction)	Classification	RNN	Precision = 99.6% and 98.7%, AUC = 0.999 and 0.996 on two test sets, respectively
[46]	mouse	Breast Tumour Vasculature	Nondestructive detection of adherent MB signatures	MB Localisation	FCN	DSC = 0.45; AUC = 0.90
[47]	pig	Spleen	Classification of splenic trauma	Classification	CNNs	Acc = 0.85; Se = 0.82; Sp = 0.88; PPV = 0.87; NPV = 0.83
[48]	pig	Heart Left Ventricle	Segmentation of left ventricle	Segmentation	CNNs	DSC = 0.90 and 0.91 for U-Net and segAN, respectively
[49]	mouse	Brain, Liver and Kidney	Segmentation of whole-body, liver and kidney	Segmentation	CNN	DSC = 0.91/0.96/0.97 for brain/liver/kidney, respectively
[50]	rat	Liver	Liver fibrosis assessment by features extraction and integration	Features Extraction	DCNN	Acc = 0.83; Se = 0.82; Sp = 0.84; AUC = 0.87 for several livers fibrosis recognition
[51]	rat	Brain Vasculature	MB tracking for mouse brain perfusion	MB Localisation	3D-CNN	↑ in resolving 10 µm micro-vessels vs. conventional approach
[52]	pig	Femoral Artery	Haemorrhage identification by exploring blood flow anomalies	Anomaly Detection	DCGAN	AUC = 0.90/0.87/0.62 immediately/10 min/30 min post-injury, respectively
[53]	rabbit	Liver	Classification of fatty liver state	Classification	CNN	Acc = 74% and 81% in testing and training data, respectively
[54]	pig	Heart	Segmentation of the heart during a cardiac arrest	Segmentation	n.a.	Borders’ recognition and tracing in porcine hearts
[55]	pig	Tooth	Identification of periodontal structures and assessment of their diagnostic dimensions	Segmentation	CNN	DSC ≥ 90 ± 7.2%; ≥78.6 ± 13.2% and ≥62.6 ± 17.7% in two test sets, for soft tissue, bone, and crown segmentation, respectively
[56]	rat	Carotid Artery	Measuring blood flow vessels with high resolution	Blood Flow Measure	CNN	↑ performance in measuring vascular stiffness and complicated flow–vessel dynamics vs. conventional techniques
[57]	mouse	Embryo	3D Segmentation and classification of embryos in normal/mutant	Segmentation + Classification	3D-CNN	DSC = 0.924/0.887 for body and BV, respectively
[58]	rat	Sentinel Lymph Node Vasculature	Improvement of lateral resolution of PA microscopy	Improvement Image Quality	CNN	↑ in resolution and signal strength and ↓ in background signal
[59]	rat	Brain Vasculature	Improving convergence rate and image reconstruction quality	Pattern Recognition	CNN	↑ performance of proposed method vs. ResNet
[60]	rabbit	Liver	Classification of liver fibrosis stages	Classification	CNN	AUC = 0.82/0.88/0.90; Se = 0.83/0.8/0.83; Sp = 0.66/0.86/0.92; Acc = 0.75/0.84/0.90 for significant fibrosis/advanced fibrosis/cirrhosis, respectively
[61]	rabbit	Spine Surface	Segmentation and 3D Reconstruction of spine surface	Segmentation	CNN	overall MAE = 0.24 ± 0.29 mm; MAE ↓ 26.28% and the number of US surface points across the lumbar region ↑ 21.61%
[62]	rabbit	Near Rectum	Removing electrical noise from the step motor to reduce scanning time	Improvement Image Quality	CNN	Good denoising
[63]	mouse	Brain Vasculature	Image Upsampling	Image Upsampling	FCN	smoother vessel boundaries, ↓ artefacts, more consistent vessel intensity and vessel profile vs. undersampled images

^1^ Abbreviations: MB: Microbubbles; PD: Power Doppler; RF: Radiofrequency; PA: Photoacustic; EMI: Electromagnetic Interference; FCN: Fully Convolutional Network; DNN: Deep Neural Network; CNN: Convolutional Neural Network; RNN: Recurrent Neural Network; DCNN: Deep Convolutional Neural Network; DCGAN: Deep Convolutional Generative Adversarial Network; AUC: Area under the receiver operator curve; Se: Sensitivity; Sp: Specificity; PLR: Positive Likelihood Ratio; NLR: Negative Likelihood Ratio; CNR: Contrast-to-Noise Ratio; Acc: Accuracy; DSC: Dice Similarity Coefficient; CTR: Contrast-to-Tissue Ratio; PSNR: Peak Signal-to-Noise Ratio; NMSE: Normalised Mean Square Error; MAE: Mean Absolute Error; PPV: Positive Predictive Value; NPV: Negative Predictive Value. ↑ indicates an increase; ↓ indicates a decrease.

**Table 2:** The main characteristics of the conference proceedings included in the analysis.

Ref	Animal Model	Anatomical District	Aim of Study ^1^	DL Network Task ^1^	DL Architecture ^1^	Main Result ^1^
[64]	dog	Left Ventricle	Tracking of left ventricle motion	Segmentation	CNN	good performance in tracking LV concerning conventional methods
[65]	pig	Femoral Vein	Detection of catheter tips	Object Detection	CNNs	classification rates of 88.8% and 91.4% and MAE = 0.279 mm and 0.478 mm for linear and phased arrays, respectively
[66]	pig	Femoral Vein	Detection of catheter tips	Object Detection	CNN	a classification rate of 91.4% and a misclassification rate of 7.86%
[67]	rabbit	Liver	Classification of fatty liver disease stages	Classification	CNN	Acc = 85.48%; Se = 91.52%; Sp = 76.67%; F1-Score = 0.89; Precision = 85.84%
[68]	rat	Brain	Visualisation of blood vessels	Improving image quality	DNN	better contrast in vascular visualisation than common methods
[69]	mouse	Liver (Hepatocellular Carcinoma)	Nondestructive detection of adherent MBs signatures	MBs detection	FCN	AUC = 0.91 and DSC = 0.56
[70]	mouse	Brain	Detection of microvessel networks	MBs detection	FCN	significant improvement in image reconstruction concerning conventional beamforming methods
[71]	pig	Lung	Detection of five lung abnormalities	Classification	CNN	Se and SP > 85% for all features except for B-lines detection
[72]	mouse	Brain, Liver and Kidney	Segmentation of whole-body, liver and kidney	Segmentation	CNN	DSC = 0.98/0.96/0.97 for brain/liver/kidney, respectively
[73]	pig	Heart	Guidewire segmentation in cardiac intervention	Segmentation	3D-CNN	MHD = 4.1; DSC = 0.56
[74]	pig	Lung	Pneumothorax detection	Feature extraction	CNN + RNN	Se = 84%; Sp = 82%; AUC = 0.88
[75]	pig	Femoral Artery	Haemorrhage identification by exploring blood flow anomalies	Anomaly Detection	GAN	Sp = 70% and Se = 81–64% immediately and 10 min post-injury, respectively
[76]	rabbit	Liver	Classification of fatty liver state	Classification	CNN	Acc = 73% on testing data compared to 60% with conventional QUS
[77]	pig	Inferior Vena Cava	Vessel Lumen Segmentation	Segmentation	CNN	DSC = 0.90; TP = 57.80; TN = 31.06; FP = 6.04; FN = 5.11 post-processing
[78]	chicken	Embryo	Improvement of image quality	Beamforming	CNN	qualitative improvements in image quality
[79]	mouse	Embryo	3D Segmentation and classification of embryos in normal/mutant	Segmentation + Classification	3D-CNN	DSC = 0.925/0.896 for body and BV, respectively
[80]	mouse	Embryo	3D Segmentation of embryo brain ventricle	Segmentation	3D-CNN	DSC = 0.896 in testing
[81]	rat	Liver	Classification of liver fibrosis severity (S0–S3)	Classification	RNN	Acc = 87.5/81.3/93.7/87.5%; AUC = 0.90/0.94/0.92/0.93, for S0/S1/S2/S3, respectively
[82]	mouse	Embryo	3D Segmentation of embryos body and brain ventricle	Segmentation	3D-CNN	DSC = 0.934/0.906 for body and BV, respectively
[83]	rat	Heart	Obtaining the position of the Epicardium and Endocardium	Segmentation	CNN	Accuracy from 82.26% to 85.03% by comparing semi-automatic with automatic segmentation method

^1^ Abbreviations: MB: Microbubbles; CNN: Convolutional Neural Network; DNN: Deep Neural Network; FCN: Fully Convolutional Network; RNN: Recurrent Neural Network; GAN: Generative Adversarial Network; MAE: Mean Absolute Error; Acc: Accuracy; Se: Sensitivity; Sp: Specificity; AUC: Area under the receiver operator curve; DSC: Dice Similarity Coefficient; MHD: Mean Hausdorff Distance; TP: True Positive; TN: True Negative; FP: False Positive; FN: False Negative.

## Data Availability

Not applicable.
